# A more tubulocentric view of diabetic kidney disease

**DOI:** 10.1007/s40620-017-0423-9

**Published:** 2017-08-24

**Authors:** Letizia Zeni, Anthony G. W. Norden, Giovanni Cancarini, Robert J. Unwin

**Affiliations:** 1 Department of Medical and Surgical Specialities, Radiological Sciences and Public Health, University of Brescia, Piazza del Mercato 15, 25121 Brescia, Italy; 2 UCL Centre for Nephrology, UCL Medical School, Royal Free Campus, Rowland Hill Street, London, NW3 2PF UK; 3 Operative Unit of Nephrology, ASST Spedali Civili, Piazzale Spedali Civili 1, Brescia, Italy; 4 Cardiovascular and Metabolic Diseases iMED ECD, AstraZeneca Gothenburg, Mölndal, Sweden

**Keywords:** Diabetic kidney disease, Microalbuminuria, Hyperfiltration, SGLT2 nephro-protection, Diabetic tubulopathy, Tubular biomakers

## Abstract

Diabetic nephropathy (DN) is a common complication of Diabetes Mellitus (DM) Types 1 and 2, and prevention of end stage renal disease (ESRD) remains a major challenge. Despite its high prevalence, the pathogenesis of DN is still controversial. Initial glomerular disease manifested by hyperfiltration and loss of glomerular size and charge permselectivity may initiate a cascade of injuries, including tubulo-interstitial disease. Clinically, ‘microalbuminuria’ is still accepted as an early biomarker of glomerular damage, despite mounting evidence that its predictive value for DN is questionable, and findings that suggest the proximal tubule is an important link in the development of DN. The concept of ‘diabetic tubulopathy’ has emerged from recent studies, and its causative role in DN is supported by clinical and experimental evidence, as well as plausible pathogenetic mechanisms. This review explores the ‘tubulocentric’ view of DN. The recent finding that inhibition of proximal tubule (PT) glucose transport (via SGLT2) is nephro-protective in diabetic patients is discussed in relation to the tubule’s potential role in DN. Studies with a tubulocentric view of DN have stimulated alternative clinical approaches to the early detection of diabetic kidney disease. There are tubular biomarkers considered as direct indicators of injury of the proximal tubule (PT), such as N-acetyl-β-D-glucosaminidase, Neutrophil Gelatinase-Associated Lipocalin and Kidney Injury Molecule-1, and other functional PT biomarkers, such as Urine free Retinol-Binding Protein 4 and Cystatin C, which reflect impaired reabsorption of filtered proteins. The clinical application of these measurements to diabetic patients will be reviewed in the context of the need for better biomarkers for early DN.

## Introduction

Diabetic Nephropathy (DN) is one of the principal long-term microvascular complications of Diabetes Mellitus (DM) Types 1 and 2. It remains a leading cause of End Stage Renal Disease (ESRD) in the United States [1] and elsewhere requiring renal replacement therapy (RRT). The rising incidence and prevalence of DM worldwide [2] creates a pressing need for earlier diagnosis and effective treatment of DM and its complications, including DN.

Initially, DN is considered to be a progressive disease characterized by significant urinary protein excretion and hyperfiltration, leading eventually to renal failure. The classical model of DN divides it into two phases, incipient and overt, which includes five progressive stages: (1) normoalbuminuria (<30 mg/g creatinine); (2) microalbuminuria (30–299 mg/g); (3) macroalbuminuria (>300 mg/g) or proteinuria (>0.5 g/g); (4) estimated glomerular filtration rate (eGFR) <30 ml/min, irrespective of albuminuria or proteinuria status; and (5) the need for renal replacement [3]. The main features of the earliest and evident stage 2 are microalbuminuria and an increase in GFR, both considered markers of glomerular damage. However, in several studies of Type 1 and Type 2 DM patients, many exceptions to this have been found. Microalbuminuria can regress (to stage 1) or remain unchanged, and a substantial proportion of diabetic individuals with decreased GFR levels are normoalbuminuric [4, 5].

There is now growing evidence that the traditional picture of DN, which is that ESRD is preceded by increasing albuminuria and proteinuria, does not always apply. Recent clinical studies have demonstrated that the ‘non-albuminuric phenotype’ is now becoming the predominant mode of DN presentation [6, 7]. While renin-angiotensin system (RAS) inhibitors have been shown to have an important action in reducing urinary albumin excretion, a positive response is not universal, and their effect may still depend more on small decreases in blood pressure and GFR. Therefore, perhaps there is another facet of nephron function, together with glomerular hemodynamics and the filtration barrier, that has a part to play in the pathophysiology of DN?

Indeed, recent findings have changed our overall concept of DN, reflected in the newer term of ‘Diabetic Kidney Disease’ (DKD) [8]. As a result of emerging evidence supporting a role for tubular involvement in DKD, interest has shifted to the proximal tubule (PT), which may play a role as an initiator, driver or contributor in the early pathogenesis of diabetes affecting the kidney.

We have conducted a literature review, firstly to address the questions of how the proximal tubule might interact with the glomerulus in early stages of DKD, and secondly to explore the evidence supporting a tubular role in initiating and/or amplifying renal damage due to DM, and lastly to determine which tubular biomarkers can potentially be used for the clinical care and follow-up of patients with diabetes. This review is not an argument against a central role for the glomerular filtration barrier or the strong evidence supporting early glomerular damage, but rather it is intended to highlight theories and evidence for PT involvement in DKD.

Although the term ‘microalbuminuria’ is widely used throughout the text, we recognize that it is potentially misleading. In fact, there is a *continuum* in the relationship between albuminuria and the risk of renal and cardiovascular disease, and the concept of a threshold level to define normality should be viewed with some caution [9].

## Microalbuminuria and hyperfiltration: an early tubular contribution to their development and a two-way interaction between glomerulus and tubule?

Microalbuminuria, a well-known marker of loss of size and charge permselectivity at the glomerulus [10], together with glomerular hyperfiltration, defined as a supraphysiologic increase in whole kidney GFR [11], highlight structural and functional changes of the glomerulus in early DKD. While it is often difficult to know what constitutes relative hyperfiltration in many Type 2 diabetics with a long history of DM and who come late to the attention of a nephrologist, there is a close interrelationship between the tubule and these classical early biomarkers of DKD.

Conventionally, the underlying mechanism of microalbuminuria in early stages of DN has been ascribed to increased glomerular filtration due to hyperfiltration or glomerular barrier damage, or some combination of the two. However, putative early involvement of the tubule, in addition to glomerular leakage, in generating microalbuminuria was highlighted in the late 1980s by clinical observations in patients with early onset Type 1 DM of relatively short duration [12]. A good correlation was found between urinary albumin excretion and markers of tubular dysfunction. Based on this finding, many animal models have been created to try to clarify the underlying mechanism. Using renal micropuncture and evaluation of endocytosis by Fluorescein-IsoThiocyanate-(FITC)-labelled albumin, and immunoelectron microscopy, a Japanese group showed in 2001 that streptozotocin (STZ)-induced diabetic rats had reduced reabsorption of albumin in proximal tubules (PT) compared with control rats. This could be partly explained by decreased albumin endocytosis with reduced megalin expression. Of importance in this study is that no increase in GFR was evident in the diabetic rats [13]. Subsequently, other groups found no significant difference in Glomerular Sieving Coefficient (GSC) between control and insulin-dependent diabetic rats, despite the presence of albuminuria in the diabetic animals. The excess urinary albumin excretion was ascribed to changes in PT albumin handling, highlighting the importance of the PT in generating albuminuria [14].

Interestingly, it has been argued that the role of the PT in determining albuminuria in renal disorders has reduced the importance of defects in the glomerular barrier. According to this ‘retrieval hypotheisis’, normal glomeruli filter high levels of albumin, which appears in the urine in nephrotic amounts only if tubular reabsorption does not occur. This means that albuminuria is primarily of tubular origin [10]. In 2007, a study performed by Russo et al. provided new insights into the contribution of post-glomerular reabsorption in excretion of urinary albumin. In their work, they reported firstly a very much higher GSC of albumin (0.034) in non-proteinuric rats than previously, and secondly they claimed that a large amount of filtered albumin underwent a rapid retrieval process via transcytosis by proximal tubule cells (PTCs); and lastly that in rats made nephrotic by purinomycin (the PAN rat model), the rate of uptake of albumin by the PT was decreased [15]. Although changes in the albumin GSC in PAN rats cannot be excluded, previous studies have demonstrated that for molecules of the same size as albumin, glomerular permeability is not altered in PAN [15, 16]. Their estimated value for GSC in rats is much higher than other measurements reported in rats. Also, a study in humans reported a much lower estimate in normal individuals, namely 8 × 10^−5^ [17]. It has been suggested that complete retrieval of the proposed amount of filtered albumin in rats with such high GSC would be unachievable [18].

Nevertheless, impaired tubular uptake and increased glomerular leakage are not mutually exclusive events [19], and both are potentially responsible for microalbuminuria in early stage DKD. Even early glomerular disease and loss of size and charge permselectivity in DM with increased albumin leakage may not cause microalbuminuria, if normal proximal tubular function can remove the excess albumin from the glomerular filtrate. Although the reserve capacity for protein uptake by the PT in humans is unknown, it is likely that the tubule does have some spare capacity for reabsorption. This suggestion is supported by the following study in the rat. Wagner et al. demonstrated that the PT regulates albumin excretion rate. In their experiments, PTs responded to an acute exogenous overload of albumin by decreasing the amount of albumin retrieved; whereas after inducing increased endogenous albumin exposure from leaky glomeruli, PTs increased the proportion of albumin taken up. So, there is some evidence for potentially large reserve capacity of PTs to reabsorb filtered albumin [20]. Dissociation between increased glomerular leakage of albumin and microalbuminuria in diabetic patients has been suggested by several clinical studies [21, 22]. In a recent study of patients with Type 2 DM, an association between PT dysfunction and podocyte biomarkers was found, which was independent of the level of albuminuria and of renal function [22].

Dickson et al. critically re-examined the steps leading to uptake and metabolism of filtered albumin in the PT and determined which stages were interrupted [19]. They highlighted the complexity of protein handling throughout the nephron. A brief summary of the studies reporting evidence of proteinuria due to tubular damage in the course of DM is presented in Table 1.

**Table 1:** Studies of tubular proteinuria and albuminuria due to tubular damage in the course of Diabetes Mellitus. Protein kinase C (PKC), sodium–hydrogen exchanger (NHE), advanced glycation end product (AGE), proximal tubular epithelial cell (PTEC)

Author, year	Findings	Possible explanation	Proposed mechanism	Experimental model
Tojo, 2001 [13]	Albuminuria in early-stage diabetic rats can be partly explained by decreased albumin endocytosis	Reduced megalin expression and increased lipid peroxidation in the proximal tubule	Uptake process	Sprague–Dawley rats with STZ-induced diabetes
Thrailkill, 2009 [23]	Megalin and cubilin were significantly higher in urine from microalbuminuric group compared with others Several megalin and/or cubilin ligands were elevated or only detected in urine from microalbuminuric group	Enhanced matrix metalloproteinase activity in the parenchyma and/or tubular lumen of the diabetic kidney may cause shedding of the megalin/cubilin complex from PT cell surfaces	Uptake process	Non-diabetic, T1DM normoalbuminuric and T1DM microalbuminuric subjects
Russo, 2009 [14]	Different distribution of endocytosed albumin in PT in diabetics *versus* controls Albumin uptake dramatically reduced in diabetic group Significant changes in peptiduria (including other proteins of filtered and cellular origin) preceded changes in intact albuminuria	Albumin handling significantly changed Peptiduria preceded changes in intact albuminuria, suggesting that this may be an earlier and more sensitive marker of DKD	Uptake process and PT handling of albumin	Type 1 diabetic Munich Wistar rats
Tojo, 2012 [24]	Albumin reabsorbed by receptor-mediated endocytosis into endosomes, where ligand-receptor dissociation must occur to recycle the albumin-binding receptors back to the plasma membrane. Vesicular acidification by H+-ATPase, chloride channel CLC-5, NHE-3 is functionally important for the pH-dependent dissociation	Renal tissue angiotensin II levels are elevated in diabetes. Angiotensin II blocks H+-ATPase, thus acidification of endosomes may be reduced by inhibition of H+-ATPase by renal angiotensin II, leading to decreased albumin reabsorption	Endocytosis process and role of Angiotensin II	Not applicable (review article)
Liu, 2015 [25]	Autophagic vacuoles are accumulated in PTECs during progression of DN Autophagy inhibition is attributed to impaired lysosomal activity after exposure to AGEs	Lysosomal membrane permeabilization and lysosomal dysfunction are triggered by AGEs Cathepsin leakage - the cytoplasmic active cathepsins released from lysosomes might trigger PTEC apoptosis Irregular lysosomal-associated membrane protein1 (LAMP1) expression Occurrence of lysosomal membrane permeabilization, which is susceptible to oxidative stress	Decrease of lysosome-mediated degradation	*In vivo* and *in vitro* model (diabetic patients and biopsy-proven DN)
Long, 2016 [26]	PT unable to dispose of internalized albumin even after several days, which led to protein engorgement. This was associated with impaired albumin uptake	PTECs have a higher capacity to take up albumin than they can process. When processing capacity is exceeded, PTECs are unable to suppress excessive uptake and accumulation of albumin continues	Processing capacity	OVE26 diabetic mouse (transgenic model of severe early-onset type 1 diabetes)

Recent findings from animal models have provided evidence for a minor contribution from other parts of the nephron to protein reabsorption, in addition to the major role of megalin-cubilin-mediated endocytosis in the PT [27]. The amount and clinical importance of these other pathways remain to be determined, particularly when glomerular ultrastructural damage and impaired PT protein uptake occur.

Regardeless of the individual contribution of either the glomerulus or PT to the quantity of urinary albumin excretion in DKD, it has been proposed that increased protein leakage from the glomerulus has intrinsic renal (tubular) toxicity [28] that may provide a pathogenic link between glomerular damage and tubulo-interstitial injury in proteinuric renal diseases. Protein overload due to impaired glomerular permeability causes excessive tubular protein reabsorption and abnormal protein accumulation in endolysosomes, which may, theoretically, up-regulate many NF-κB (nuclear factor kappa-light-chain-enhancer of activated B cells)-dependent or -independent inflammatory genes (chemokines, cytokines and endothelin) in the PT epithelium. These are potentially capable of triggering an interstitial inflammatory reaction. Consequently, the proximal tubule has a profibrotic and proinflammatory role leading to synthesis and deposition of extracellular matrix, and contributing to renal scarring [28]. Interestingly, Nielsen et al. have showed that in an FSGS (Focal Segmental GlomeruloSclerosis) mouse model, there is a counteracting mechanism against protein overload by the PT. Inflammatory mediators originating from PTECs during protein overload occurred early in the course of exposure. However, the lysosomal system showed a well-adapted response to protein load by increasing synthesis of factors for lysosomal proteolysis. Are there any other injury sensors that might stimulate inflammation and fibrosis in the PT in response to protein overload [29]? Although the importance of proteinuria as a marker and driver of kidney disease has been examined in many proteinuric renal disorders, a study by Guo et al. showed, interestingly, that tubular injury is dependent only on the degree of podocyte damage and not on albuminuria *per se* [29, 30].

## Hyperfiltration: more than a vascular effect?

Despite difficulties in a precise definition or threshold, increased GFR as a marker of glomerular hyperfiltration occurs early in the clinical course of DKD and is considered to be an important factor in the initiation and progression of kidney damage. It is thought to be due to a rise in intraglomerular pressure (causing barotrauma) and renal blood flow, resulting from an imbalance of vasoactive humoral factors that control pre-and post-glomerular arteriolar tone [11].

Clearly, a rise in hydrostatic pressure in the glomerulus may result in higher filtration pressure with an increase in the amount of protein passing through the glomerular barrier [11]. Therefore, the amount of protein delivered to the PT per unit time for potential reabsorption increases with hyperfiltration.

As discussed in a recently published review, the pathogenesis of glomerular hyperfiltration is complex and includes overlapping mechanisms, as summarized in the ‘Tubular Theory’. This theory proposes that several tubular changes inhibit tubuloglomerular feedback (TGF). This negative feedback mechanism is normally controlled by sodium and chloride delivery to the *macula densa* of the loop of Henle. In diabetes with hyperglycemia, increased filtered glucose stimulates PT glucose reabsorption that is coupled to sodium, thereby reducing its delivery to the *macula densa*, and decreasing TGF feedback. This results in afferent arteriolar vasorelaxation, increased renal blood flow and hyperfiltration [31]. Interestingly, a study exploring the effect of glomerular hyperfiltration on tubular dysfunction reported that two markers of tubular damage (Neutrophil gelatinase-associated lipocalin NGAL and Kidney Injury Molecule-1 KIM-1) showed increased excretion in the urine of those with glomerular hyperfiltration and it correlated positively with GFR. These findings suggest that glomerular hyperfiltration is related to altered tubular function in DM patients [32].

Crucially, a study by Vallon et al. showed that inhibition of PT glucose reabsorption increased the amount of sodium delivered to the *macula densa*, stimulating TGF and reducing glomerular hyperfiltration, without a change in blood glucose concentration. The tubular hypothesis of diabetic glomerular hyperfiltration suggests that the PT has a role in determining hyperfiltration, in addition to blood glucose control, through glucose reabsorption [33].

As well as increased glucose overload in the course of DM, parallel tubular hypertrophy and upregulation of sodium-glucose cotransporter 2 (SGLT2) and sodium-hydrogen exchange (NHE) 3 may also contribute to increasing hyperfiltration. Furthermore, enhanced PT reabsorption may reduce intratubular pressure and so increase the net pressure gradient for filtration across the glomerulus [11].

**Fig. 1:**
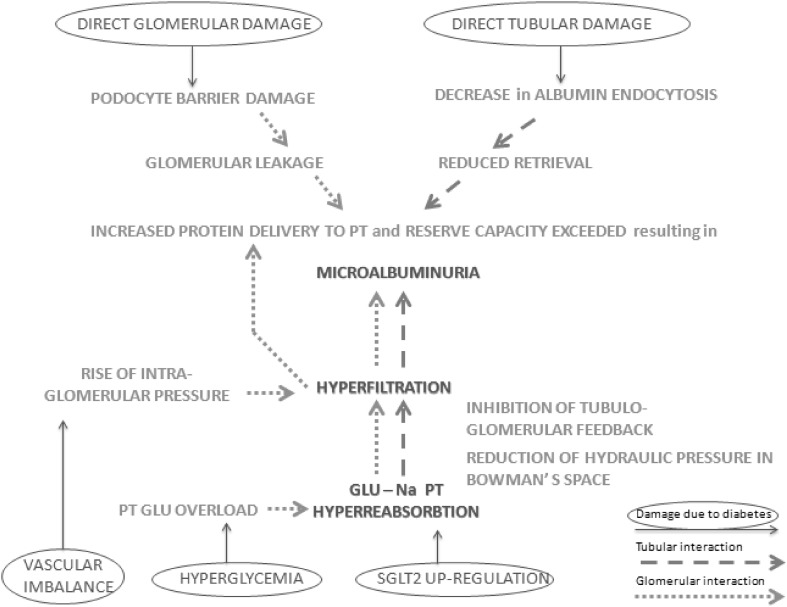
Interactions between glomerulus and proximal tubule (PT) in the pathophysiology of microalbuminuria, hyperfiltration and proximal tubule glucose (GLU) reabsorption in the context of DKD

Understanding the relationship between damage to different structures in the nephron and determining which lesions are primary is a major challenge (Fig. 1). However, there appears to be good evidence for early tubular involvement in the course of DKD and its role in the pathogenesis of both microalbuminuria and hyperfiltration.

## The proximal tubule: a therapeutic target for nephro-protection

In normoglycemic humans, the high-capacity cotransporter SGLT2 is responsible for some 97% of glucose reabsorption by the PT, and retrieves filtered glucose with sodium in a 1:1 molar ratio; SGLT1 in the later part of the PT reabsorbs any remaining luminal glucose. The two basolateral glucose transporters, GLUT1 and GLUT2 can return reabsorbed glucose to the bloodstream. Different structures within the PT co-operate to help to maintain the blood glucose level [34].

The glucose load in PT is increased with hyperglycemia in diabetes and exceeds the renal transport maximum for glucose, leading to glycosuria in poorly controlled DM. Blocking glucose reabsorption in the PT increases glucose excretion in the urine and can reduce high circulating blood glucose levels. Canagliflozin is an example of a SGLT2 inhibitor that was approved by the Food and Drug Administration (FDA) in 2013 as a new glucose-lowering medication for adults with type 2 diabetes.

Use of SGLT2 inhibitors has brought new insights into the complex mechanism of PT glucose reabsorption. Previously, only medication blocking RAS showed some nephro-protective effects in patients with DM. New therapeutic strategies to improve renal outcome have been sought and in a recently published study Empagliflozin, another selective SGLT2 inhibitor, significantly (and unexpectedly) slowed progression of DKD and lowered the risk of clinically relevant renal events in type 2 DM patients [35], as well as heart failure. This new class of drug appears very promising. In addition to the EMPA-REG study, there are several ongoing studies looking at the effects of SGLT2 inhibition on cardiovascular and kidney outcomes, including DECLARE, CANVAS, and CREDENCE [36].

Why does targeting the SGLT2 cotransporter improve renal outcomes in addition to many metabolic and cardiovascular complications in DM? Current thinking is that the beneficial effect of SGLT2 inhibition is primarily a direct nephro-vascular effect [31]. Activation of TGF is a myogenic response to distal sodium delivery, inducing glomerular arteriolar vasoconstriction and reduction in GFR [31]. DM with hyperglycemia is associated with renal sodium retention and increased proximal tubular reabsorption, reducing distal delivery and decreasing TGF, and thus hyperfiltration [34]. Therefore, the beneficial renal effect of Empaglifozin highlighted in the EMPA-REG OUTCOME Trial is thought to be the consequence of reducing intraglomerular pressure and barotrauma to podocytes [31]. This mechanism may also underlie the associated decrease in urinary albumin excretion seen with SGLT2 inhibition [37].

Despite the reduction in glomerular hyperfiltration and podocyte stress, SGLT2 inhibitors have other potentially nephro-protective mechanisms that may be renal or systemic. Looking at the former, some important pathophysiological changes in the nephron have been reported in relation to DM [34]:

- Upregulation in renal SGLT2 expression has been demonstrated in both human cells and some animal models of Type 1 and Type 2 DM [34]. It is still unclear whether this phenomenon is the result of hypertrophy of PT in DM and/or of an ‘energy/glucose saving’ response to a glucose sensor downstream of the early proximal tubule. This maladaptive process of SGLT2 upregulation helps to sustain high blood glucose. Therefore, targeting this upregulated cotransport specifically, means a better glycosuric and blood glucose-lowering effect and, perhaps, also a prevention of glucose toxicity to PTEC [33].
- Upregulation of SGLT2 has been demonstrated in both human and animal models of type 1 and type 2 DM, but what happens to SGLT1 activity is more controversial. Under high glucose conditions, SGLT1 expression has been reported to be increased, reduced or unchanged. In STZ-diabetic rats and in diabetic obese Zucker rats an increase in SGLT1 mRNA expression was documented, whereas in Akita mice models of type 1 DM, as well as in normoglycemic rats under pharmacological or genetic SGLT2 inhibition, SGLT1 renal protein expression decreased. Thus, these animal studies show contrasting changes in SGLT1 levels in different rodent models of diabetes [38]. When SGLT2 function is saturated by glucose overload due to DM or, alternatively, is inhibited pharmacologically or genetically, does SGLT1 increase its activity to prevent a loss of glucose in the urine? Rieg et al. showed that with genetic or pharmacological inhibition of SGLT2 in non-diabetic mice, a compensatory increase in SGLT1-mediated transport occurs, which explains why renal fractional glucose reabsorption is maintained at 40–50% [39]. Following this reasoning, Abdul-Ghani et al. [40] in 2013 suggested that SGLT2 inhibitors cause SGLT1 to reabsorb glucose at its maximum capacity, unmasking its global reabsorption reserve, in accordance with the results of Powell et al. [41]. The latter group demonstrated that in SGLT2 knockout mice urine glucose excretion is approximately 30% of filtered glucose. Consistent with this, observations in humans showed a fractional glucose reabsorption rate of about 40–50% following a maximal effective dose of SGLT2 inhibitor. A compensatory effect of SGLT1 is also supported by micropuncture studies along the late proximal tubule in Sglt2−/− mice [38]. Thus, there is an increase in glucose reabsorption mediated by SGLT1 when the principal cotransporter (SGLT2) is functionally deleted. Therefore, when a compensatory increased activity of SGLT1 occurs in response to an excessive glucose to be reabsorbed, one would expect an upregulation of this tubular transporter. However, in a genetic model of T1DM (Akita mice) and in response to SGLT2 gene knockout and pharmacological inhibition of SGLT2 in non-diabetic mice, SGLT1 protein expression was found to be reduced. How can this apparent discrepancy be explained? Downregulation of SGLT1 expression, would attenuate any increase in glucose reabsorption and promote glycosuria, which has been put forward as protective mechanism for the kidney. The late PT is highly vulnerable to hypoxia and acute injury in general, and Vallon et al. proposed that downregulation of SGLT1 expression could mitigate or prevent glucose toxicity in this nephron segment [42].
- Rat studies suggested the translocation of GLUT2 from basolateral to apical brush border PT membrane in diabetes [43]. A similar mechanism occurs in the small intestine, where SGLT1 sensitivity is required for GLUT2 translocation. Does SGLT2 in the kidney play the same role of sensor that SGLT1 has in the small intestine to allow GLUT2 translocation? Furthermore, does the translocation occur even if SGLT2 is pharmacologically inhibited? Moreover, it is still unknown whether the translocation of GLUT2 occurs in humans and if a GLUT2 contribution in facilitating glucose reabsorption when PT lumen is exposed to high glucose concentration is physiologically relevant [34].

Recent evidence suggests a functional link between SGLT2 and the sodium–hydrogen exchanger (NHE) 3 in that SGLT2 inhibition may also inhibit NHE3 in the proximal tubule. Taking all the evidence together, it is suggested that the nephro-protective effects of SGLT2 inhibitors are directly or indirectly related to decreased sodium reabsorption. However, an alternative explanation has been proposed [44]. Firstly, Wakisaka suggested that SGLT2 inhibitors may increase sodium reabsorption in the proximal tubule [45], in contrast to the still widely accepted explanation for SGLT2 inhibition and nephro-protection. However, the reasoning is that in the course of SGLT2 inhibition, SGLT1 has a greater role in glucose reabsorption with a glucose to sodium ratio of 1:2, rather than 1:1 with SGLT2. Secondly, Wakisaka proposed that SGLT2 inhibition and nephro-protection is not dependent entirely on TGF. In fact, in the 1990s the presence of SGLT2 and GLUT1 was also recognised in glomerular mesangial cells. Given that the loss of mesangial contractility is hypothesized to be another potential mechanism for glomerular hyperfiltration, Wakisaka explored the effect of high glucose concentration on mesangial cells. After incubation of rat mesangial cells for 6 days in 20 mM glucose, the contraction of mesangial cells in response to angiotensin II showed a decrease. This mechanism was ascribed to SGLT2 acting as a glucose sensor and sodium-calcium exchanger, because Phlorizin, a competitive inhibitor of SGLT1 and SGLT2, attenuated mesangial cell dysfunction, normalizing its contractile response [46]. The author concluded that the nephro-protective effect of SGLT2 inhibition was partially driven by its direct cellular action on mesangial cells, offering a new perspective [45].

The kidney has a complex structure–function relationship and nephro-protection is likely to depend on several mechanisms yet to be elucidated. In this sense, many pleiotropic effects of SGLT2 inhibition have been postulated. Given that the PT is an actively transporting segment of the nephron, with a high number of transporters requiring a continuous energy supply, and with a large number of mitochondria in PTECs, we should consider the ‘thrifty substrate hypothesis’ in this context. According to this hypothesis, SGLT2 inhibition through mild ketosis promotes β-hydroxybutyrate uptake in the myocardium and kidney. This substrate selection improves mitochondrial efficiency and ATP generation. In addition, the modest hemoconcentration that follows SGLT2 inhibitor treatment may increase tissue oxygen delivery [47]. Another mechanism that also might contribute to nephro-protection of SGLT2 inhibitors is the increased renal content of HIF1-α [42].

Is the downregulation of SGLT1 expression a further nephro-protective mechanism? Could the sodium-glucose cotransporter SGLT1 be a second renal therapeutic target in DKD? Although this has been considered, its main target, and potential source of benefit, would be inhibiting intestinal absorption of glucose and postprandial excursions in blood glucose. How significant for nephro-protection is any effect on mesangial cells? Are there additional effects of SGLT2 inhibitors that explain the positive renal outcome in DM patients? Considering the evidence as a whole, renal glucose reabsorption is ‘dysregulated’ in DM and the data so far suggest that several mechanisms may underlie nephro-protection by SGLT2 inhibitors. More studies are needed to understand better how the PT handles glucose in DM.

## Diabetic tubulopathy: from pathophysiological mechanisms to histological data and evidence for a causative role for the renal tubule in DKD

The interplay of the glomerulus and proximal tubule in DKD has been described in the pathogenesis of proteinuria, hyperfiltration and, importantly, nephro-protection. Nevertheless, there are findings that suggest a primary role for the PT in causing nephron injury and dysfunction, defined by the term ‘Diabetic Tubulopathy’ [48]. Because of their position and major reabsorptive role within the nephron, PTECs are exposed to factors in the glomerular filtrate, in the peritubular capillary blood supply, and in the interstitium. Consequently, they can be injured by a variety of potentially damaging agents that can trigger a pro-inflammatory and pro-fibrotic response causing tissue injury. In 1999, Gilbert and Cooper highlighted non-glomerular mechanisms involved in tubular cell damage in diabetes, emphasising that tubulo-interstitial injury was more than could be explained by glomerular injury alone [49].

The factors directly related to glucose metabolism, and potentially implicated in determining the pro-inflammatory and pro-fibrotic phenotype of the renal tubule in DKD, are a high glucose concentration and formation of advanced glycosylation end-products (AGEs). A summary of the main mechanisms thought to be involved is shown in Table 2. Why are PTECs so vulnerable to glucose damage? PTECs are exposed to glucose apically from the filtered glucose load or basolaterally through elevated interstitial tissue concentrations of glucose. PTECs cannot decrease glucose transport to prevent excessive changes in intracellular glucose when exposed to high glucose concentrations [50].

**Table 2:** Mechanisms of damage due to high glucose concentration and advanced glycosylation end-products (AGEs) on the renal tubule in diabetes mellitus

Stimulus	Injury pathway	Effects on proximal tubule
High glucose	Increased expression of the pro-fibrotic cytokine TGF-β	Production of collagens type I and type IV with autocrine and paracrine effects on interstitial cells [49]
	Acceleration of polyol pathway metabolism and accumulation of sorbitol	Stimulation of extracellular matrix expression [49]
	Increased glucose uptake induces angiotensin II, TGF-β and cyclin-dependent kinase inhibitors	Cell cycle arrest and a switch to tubular hypertrophy and a senescence-like phenotype [51]
	Promotion of angiotensinogen and AT1 expression	Increased TGF β1 expression and PTECs hypetrophy oxidative stress [49]
	Production of VEGF, TGF β, IL-6, CCL-2 partly through MAPK, PKC signalling, TLR	Neo-angiogenic, pro-fibrotic and pro-inflammatory PTECs shift [48]
	Upregulation of MIP-3α	Intracellular oxidative stress [48]
	KLF6 over-expression and activation of p38 signaling and activator protein-1	Promotion of epithelial mesenchymal transition [48]
	Generation of intracellular (mitochondrial) ROS	Reduction of NO and vasoconstriction of peritubular vessels. Pro-inflammatory gene upregulation. Oxidative stress [52]
	SGK-1 overexpression	Increased proximal tubular cell growth, progression through the cell cycle, and inhibited apoptosis [50]
	Inhibition of hypoxia-induced activation of HIF and VEGF expression	Reduced protection of hypoxic tissues [50]
Advanced glycosylation end-products (AGEs)	Activation of intracellular second messenger mitogenic activated protein kinase	Increased TGF β1 expression [49]
	Increased in cytosolic phospholipase A2 α activity and cellular phosphoinositol 4,5 bisphosphate production	Generation of intracellular ROS and Oxidative stress [52]
	Increased circulation and therefore increased catabolism. Increased PT AGE binding→ Stimulation of IL-8 and ICAM-1 expression via NF-κB, MAPK- and STAT-1-dependent pathways and TBM glycation	Infiltration of Leukocytes [48]
	Upregulation of tubular expression of CTGF, TGF β, VEGF. Stimulated expression of IL-6 and CCL-2. Activation of NF-κB	Neoangiogenetic, profibrotic and proinflammatory PTEC shift [51]

Proximal tubule (PT) Transforming growth factor beta (TGF β), Angiotensin II receptor type 1 (AT1), Vascular endothelial growth factor (VEGF), chemokine (C-C motif) ligand 2 (CCL-2), Mitogen-activated protein kinase (MAPK), Protein kinase C (PKC), Toll like receptor (TLR), Macrophage Inflammatory Protein-3 MIP-3α, Krueppel-like factor 6 (KLF6), serum and glucocorticoid-regulated kinase 1 (SGK-1), Hypoxia-inducible factors (HIF), Intercellular Adhesion Molecule 1(ICAM- 1), Signal transducer and activator of transcription 1 (STAT- 1), connective tissue growth factor (CTGF)

However, there are other stimuli, not strictly related to glucose control that may affect the proximal tubule, for example, activation of local vasoactive hormone systems that cause hemodynamic changes. Over-expression of endothelin [49] and local angiotensin II in the renal tubule, activation of vascular endothelial growth factor (VEGF), and reduction of nitric oxide (NO) [52, 53] may cause vasoconstriction of the afferent and efferent arterioles, as well as the interlobular arteries. The combination of several vascular and metabolic effects may induce ischemic damage to PTECs. This may cause elevated energy demand of tubules due to increased reabsorption of glucose, AGEs and proteins, as well as reductions in peritubular blood flow with post-glomerular vasoconstriction and capillary loss following tubulo-interstitial expansion [49]. In addition, elevated blood pressure, often associated with impaired glucose control, may induce mechanical stress in the peritubular capillary network, which compared with the glomerulus is poorly adapted to cope with an increased blood pressure [49] and mechanical shear stress. All the changes in the vascular factors mentioned above can lower post-glomerular blood flow in diabetes and contribute to further reductions in oxygen delivery to the tubules and hypoxia.

As well as hemodynamic changes, other pathways are likely to contribute to the pathogenesis of tubular damage in DKD. Purinergic signalling may be involved, since it has been shown that this pathway is part of an inflammatory cascade and can lead to renal glomerular, tubular and vascular cell damage in a variety of inflammatory renal diseases, including DKD. Local productions of chemokines, adhesion molecules, and inflammatory cytokines are upregulated by chronic stimulation from hyperglycemia and other modulators [54]. In type 2 diabetic patients with nephropathy, tubular P2X4R (P2X4R purinoreceptor) expression is upregulated and closely related to NLRP3 inflammasome activation and renal interstitial inflammation [55].

Recent studies have also shown the importance of the endocannabinoid system in normal PTC function. In the model of Jenkin et al., increased circulating endocannabinoids in DM modify the expression of cannabinoid receptors in PTECs that might participate in activation of inflammation and cell hypertrophy, as well as in tubular cell dysfunction [56]. In DKD, the purinergic and endocannabinoid systems deserve further investigation as potential targets for therapeutic intervention. Finally, there are a number of pro-inflammatory factors abnormally filtered by altered glomeruli in the course of diabetes that could disrupt PTEC function, including IL-6, Il-8, IL-1β, ROS, MCP-1 and RANTES [57].

Given the uncertainty and speculation over its pathogenesis, is diabetic tubulopathy a real entity in DKD and does it have any implications for treatment? A close correlation between the extent of tubulo-interstitial injury and long-term renal function in a variety of primary glomerular diseases has been demonstrated [49]. Furthermore, the extent of renal dysfunction is generally poorly associated with changes in glomerular morphology, whereas it correlates well with chronic tubulo-interstitial injury [48]. A recent review of the histological changes in early DKD described tubular cell hypertrophy, thickening of the tubular basement membrane, and interstitial inflammation with mononuclear cell infiltration. Progression of these early tubular abnormalities leads to interstitial fibrosis and tubular atrophy (IFTA) [58].

Brito et al. reported that in T1DM, the tubular cell basement membrane (TBM) width showed a strong correlation with glomerular basement membrane (GBM) width and mesangial expansion, the two most important and characteristic findings in the glomeruli of DKD. In the same report, TBM and GBM width were more closely correlated with glycosylated haemoglobin than other renal structural measures [59]. Moreover, the first measurable change, GBM thickening, has been detected very early in the course of DKD within 1.5 to 2.5 years after onset. Therefore, the parallel change in TBM may also occur very early and is a primary abnormality, and it is not secondary to glomerular hemodynamic changes [60].

Furthermore, early changes have been found in the PT in the rat model of Streptozotocin-induced diabetes: an early hyperplastic phase, followed by either transition to hypertrophy or cellular senescence. An accelerated senescent phenotype was also found in tubular cells of T2DM patients with nephropathy [61]. PTECs show increased lipofuscin pigment and vacuolarization, perhaps due to a high tubular lysosomal load and cell adaptation to stressful stimuli such as hyperglycemia, glycogen accumulation and subnuclear lipid vacuolarization [58].

In the later stages of DKD in both T1DM and T2DM patients, abnormalities involving the glomerulotubular junction have been described, with a glomerulus without a tubular attachment and known as an ‘atubular glomerulus’ (AG), which is non-functioning, or an atrophic tubule [60]. Najafian et al. showed that the fractional volume of atrophic tubules, percent of atubular glomeruli, and percent of glomeruli with tip lesions accounted for 94% of the GFR variability in diabetic patients, highlighting the importance of AG and glomerulotubular junction abnormalities in the development and progression of DN [62].

## Tubular biomarkers: use in clinical care

The significant incidence and prevalence of DKD across diabetic populations and the limitations of microalbuminuria have emerged recently. Novel and better biomarkers for clinical diagnosis and management of DKD are needed to provide earlier diagnosis and more accurate prognosis. Microalbuminuria does still have a useful role in screening diabetic patients for DKD. It has several advantages: it is organ specific and a marker of generalized endothelial dysfunction with important prognostic implications for cardiovascular and renal outcomes [63], and measurements are widely available. It also reflects glomerular injury, as well as tubular dysfunction. However, its evolution is not predictable: microalbuminuria can regress towards normal values, progress towards macroalbuminuria, or remain stable for many years [64]. Moreover, there is evidence that DKD can develop with normoalbuminuria, and even though microalbuminuria is currently first-line screening for kidney involvement in DM, structural changes may appear in the glomerulus before it develops [65].

**Fig. 2:**
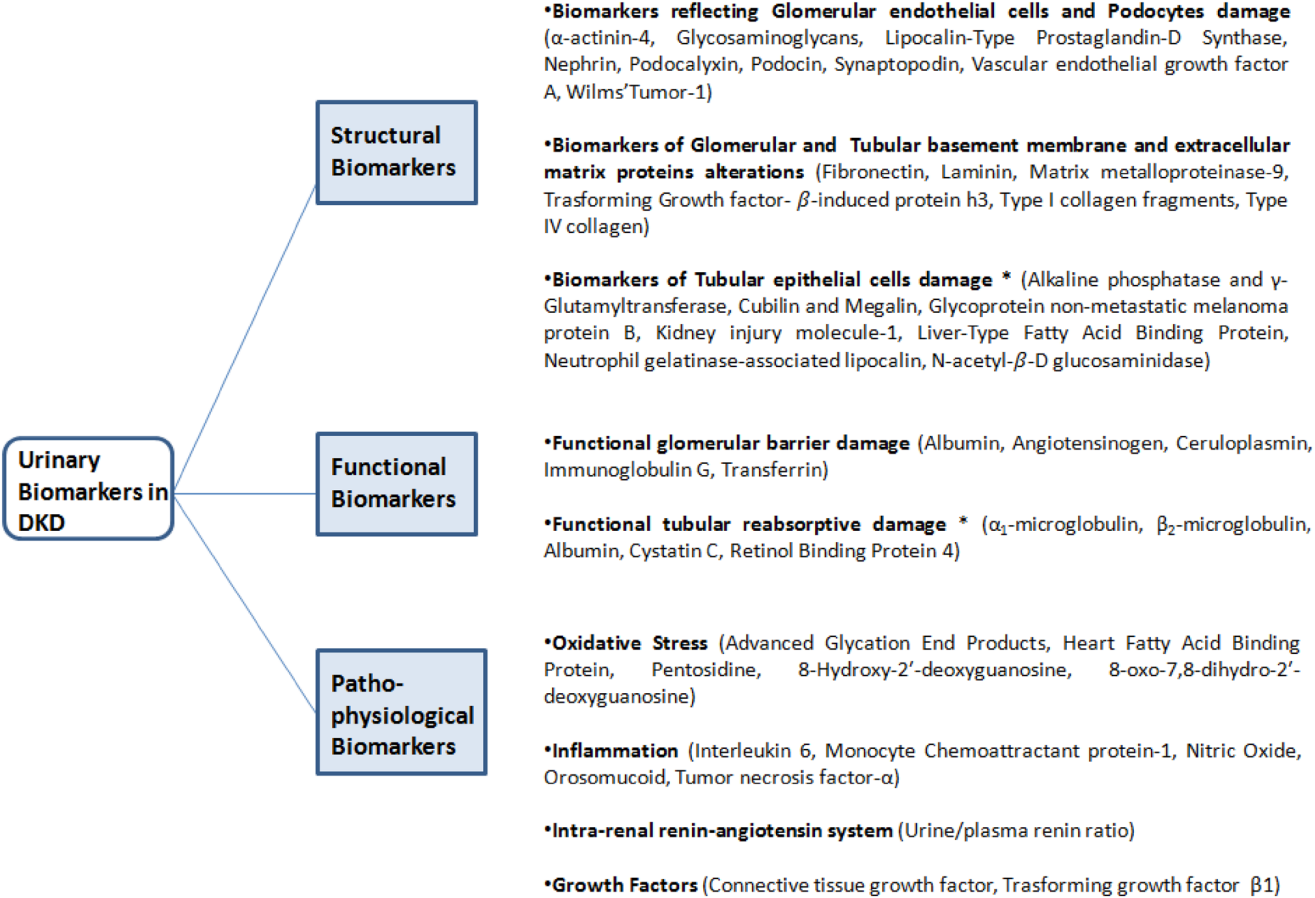
Classification of urinary biomarkers in diabetic kidney disease [64–66]. *Tubular biomarker further discussed in the text

To overcome the limitations of microalbuminuria, new biomarkers in DKD are being sought. A clinically useful biomarker needs to be detectable early in the pathophysiological process of a disease, to have high sensitivity and specificity, and to provide high diagnostic and prognostic values. There are several novel and promising urinary biomarkers for renal damage in the early stages of DKD [64–66]. Urinary biomarkers in DKD can indicate the site of damage in the nephron, impaired function of the nephron, or the underlying pathophysiological process. We summarize these urinary biomarkers in DKD in Fig. 2, which outlines the principal tubular biomarkers that could be helpful in early detection of DKD.

What measurements are appropriate for detecting PT dysfunction in DKD? There are both functional and structural markers. One approach is to evaluate PT dysfunction by assessing impaired reabsorption of filtered proteins. The major site for filtered protein reabsorption is in the PT and assuming no significant post-glomerular degradation or secretion of these proteins, theoretically the more freely filtered the protein is, the greater should be the increment in urinary excretion when the tubular reuptake process is disrupted. In this category of ‘Functional Tubular Biomarkers’ (FTB) are included plasma proteins of low molecular weight (LMWP) that are freely filtered by the glomerulus and almost fully reabsorbed by the PT. Another approach is to identify those substances excreted in urine as a result of tubular damage or regeneration. These ‘Structural Tubular Biomarkers’ (STB) are urinary enzymes that originate not from plasma, but directly from tubular cells.

In ‘tubular’ proteinuria, PT endocytic function is impaired and large amounts of LMWP appear in the urine, for example, UfRBP4 is elevated some 1,000-fold when endocytic function is completely abolished, as occurs in a number of monogenic tubular diseases [18]. The conventional explanation for increased excretion of LMWP is tubular disease. However, there is another possible mechanism of increased LMWP excretion in DM. There is evidence from at least one animal model that proteins compete for reabsorption by the proximal tubule [67]. There is also clinical evidence that the same pathway is used for uptake of filtered albumin and LMWP in humans, suggesting that two or more proteins might compete for reabsorption [68]. This would have the effect of increasing the excretion of a freely filtered plasma protein as a result of increased filtration of other proteins, such as albumin. This might occur when the size and charge permselectivity of the glomerulus is impaired in DKD. However, even early glomerular disease and loss of size and charge permselectivity in DM with increased albumin leakage may not cause microalbuminuria, if normal tubular function can reabsorb the excess albumin from the glomerular filtrate. The reserve capacity for protein uptake by the proximal tubule is unknown, but it is likely that the tubule has some reserve capacity for reabsorption; however, competition for reuptake between albumin and LMWP, as discussed earlier, may occur. Consequently, small increases in albumin leakage across the glomerulus in early DM may not cause ‘microalbuminuria’, but would be detected indirectly by measurement of increased LMWP excretion. Others have also proposed dissociation between increased glomerular leakage of albumin and microalbuminuria [22]. A possible limitation of the use of functional tubule biomarkers is an altered level in the serum (plasma overload) and/or alteration of glomerular filtration rate.

The principal tubular biomarkers in DKD are briefly described in Table 3. The major strengths of several of them are [64–66]:

- a good correlation between the degree of damage at tubulo-interstitial level and the deterioration of renal function
- an early detection of tubule damage in the course of DKD
- tubular involvement in both T1DM and T2DM
- presence in normoalbuminuric patients
- a correlation with duration, severity and control of DM
- a progressive increase in patients with micro and macroalbuminuria.

**Table 3:** Principal structural and functional tubular biomarkers over-expressed in the urine and explored in clinical background of diabetic kidney disease [18, 64–66, 69]. Proteins of Low Molecular Weight (LMWP), Proximal tubule (PT), Proximal Tubule Epithelial Cells (PTECs), Molecular weight (MW), brush border (BB)

Functional tubular biomarkers (FTB)	
Retinol-binding protein 4	LMWP (~21 kDa when not bound to transthyretin), freely filtered by the glomerulus and almost completely reabsorbed in the PT. No tubular secretion. Measurement of free form of uRBP4 performs significantly better than previous measurement of total uRBP4 in the discrimination of patients with proximal renal tubular disorders
Cystatin C	Cysteine protease inhibitor with MW 13 kDa freely filtered by the glomerulus and almost entirely reabsorbed in the PT. No tubular secretion
α_1_-microglobulin	Glycoprotein with MW 26–31 kDa. The unbound form is filtered freely through the renal glomerular basement membrane and is reabsorbed by the PTECs. No tubular secretion
β_2_-microglobulin	LMWP (11.8 kDa) filtered by the glomerulus and is degradated in the PT via a megalin-dependent pathway. Unstable in urine
Albumin	Molecular weight of 65 kDa; normally very little is filtered at the glomerulus. With glomerular barrier damage, filtration occurs and is followed by tubular reabsorption; the resulting albuminuria reflects the combined contribution of these two processes. Reserve capacity for reabsorption by PT is unknown
Structural tubular biomarkers (STB)	
Neutrophil gelatinase-associated lipocalin (NGAL)	A 25 kDa protein covalently bound to gelatinase from human neutrophils and part of the lipocalin family. NGAL is hyper-produced in the kidney tubules within a few hours after insults such as ischemia–reperfusion. It is freely filtered and reabsorbed in PT. Although it can be regarded as both FTB and STB, it is mainly considered a STB
Kidney injury molecule-1 (KIM-1)	A type 1 transmembrane protein expressed on the apical membrane of PT cells. Its ectodomain is cleaved and released into the lumen of the tubule and ultimately appears in the urine. KIM-1 facilitates repair of the damage by removing cellular debris and apoptotic bodies from the injured tubulo-interstitial compartment. Elevated in acute kidney damage
N-acetyl-β-D glucosaminidase (NAG)	Lysosomal brush border enzyme found in the PT cells. Because of its relatively high molecular weight (>130 kDa), plasma NAG is not filtered though the glomeruli. NAG is released into the urine after renal tubule injury
Liver-type fatty acid binding protein (L-FABP)	Intracellular carrier protein expressed in the cytoplasm of human PT cells. MW: 14.2 kDa. Believed to have protective functions. Its excretion is associated with structural and functional tubular damage. Moreover, it is freely filtered and reabsorbed in PT. Although it can be regarded as both FTB and STB, it is mainly considered as a STB
Cubilin and Megalin	Two apical membrane receptors responsible for endocytosis via clathrin-coated vesicles, the central mechanism for protein reabsorption in the PT. Megalin is an approximately 600 kDa transmembrane protein belonging to the LDL receptor family, and Cubilin is a slightly smaller peripheral membrane protein, approximately 460 kDa. Most proteins filtered through glomeruli have been identified as ligands of megalin, cubilin, or both
Alkaline phosphatase (ALP) and γ-Glutamyltransferase (GGT)	ALP is an enzyme with an MW 70–120 kDa. It is associated with the membranes of cell surfaces located in the PT, especially in the BB of epithelial cells. It originates from damaged renal tubules, and its levels are associated with the degree of damage. GGT is an enzyme with a molecular weight ~90 kDa. It is present in the PT and the increased GGT excretion in the urine reflects the damage of the BB membrane and the loss of microvilli. The urinary levels of these enzymes/proteins are not influenced by their plasma levels
Glycoprotein non-metastatic melanoma B (Gpnmb)	A transmembrane glycoprotein expressed on renal tubular cells. Increased during repair after renal ischemia—reperfusion injury. It may be a marker of tubular regeneration. Elevated in proteinuric renal diseases including diabetic nephropathy

The main limitation of tubular biomarkers is their poor independent predictive value for GFR decline and development of albuminuria, although there are conflicting findings in relation to their predictive value. One study assessed two tubular damage biomarkers, N-acetyl-β-D glucosaminidase (NAG) and β_2_ microglobulin. They did not add any prognostic benefit in detecting progressive renal impairment defined as a decline in eGFR of ≥50% from baseline, or start of dialysis, in T2DM, whereas histological findings of tubular atrophy and interstitial fibrosis (IFTA) did; although both NAG and β_2_ microglobulin significantly correlated with the severity of tubulo-interstitial lesions. In fact, the IFTA scores were good predictors of renal prognosis, independent of other indicators of progression [70]. However, this was a retrospective analysis of over 25 years and over such a long period it is likely that both the sensitivity and accuracy of biomarker measurements have changed, as well as clinical care. A 3-year prospective intervention trial by Nielsen et al. found that T1DM patients with high levels of urinary NGAL and KIM-1 had a faster decline in GFR, suggesting that tubular damage is important for progression, even though the markers did not add to other markers of likely progression [71]. A limitation of this study was the storage of samples at only −20 °C for 10 years before analysis. Tubular biomarkers are sensitive to their handling and storage conditions, and even −70 °C can degrade over several years [72]. In a study by Conway et al. uKIM-1/Cr and uGpnmb/Cr ratios were elevated in patients with incipient DN due to T2DM, suggesting an ongoing tubular injury. Both tubular biomarkers were correlated with the severity of proteinuria and with a faster decline in renal function. Nonetheless, neither uKIM-1/Cr nor uGpnmb/Cr ratio added significant prognostic value to ACR alone in a 4-year-follow up. Perhaps in can take many years before low-grade tubular injury translates to a decline in eGFR [73].

Nevertheless, there are other studies reporting that some tubular biomarkers do have a role in predicting the evolution of DKD. In 2010, Kern et al. showed that the baseline excretion of NAG and its increase over time independently predicted both micro- and macroalbuminuria in T1DM patients [74]. In 2011 Fu et al. in a study of T2DM patients and healthy controls showed that NGAL increased significantly across the four groups from controls to normoalbuminuric, microalbuminuric and macroalbuminuric patients. In addition, NGAL as well as NAG, correlated with the urinary albumin-creatinine ratio (UACR) in the normoalbuminuric group, potentially predicting microalbuminuria and demonstrating a negative correlation with eGFR in the macroalbuminuria group. Furthermore, recent studies have explored the predictive value of tubular biomarkers in DKD [75]. In 2012, Soggiu et al. demonstrated increased retinol -binding protein 4 (RBP4) and α_1_-microglobulin in microalbuminuric patients. These results also showed a positive correlation between increases of LMWP and albuminuria. Thus, increased excretion of RBP4 and α_1_-microglobulin could be predictive of early-stage nephropathy in T1DM [76]. Recently, Panduru et al. showed that in a large cohort of patients with T1DM with a median follow up of 5.8 years, Liver-Type Fatty Acid Binding Protein (L-FABP) was an independent predictor of DKD evolution as assessed by progression from normo- to microalbuminuria, from microalbuminuria to macroalbuminuria, and from macroalbuminuria to ESRD [77]. A Korean group has also published results of 237 T2DM patients enrolled from May 2008 to December 2009, followed annually until March 2012 and screened for Cystatin C and non-albumin protein (NAP). Both measurements were significantly associated with a decline in eGFR after adjusting for age and several clinical confounders [78].

To clarify their utility in the clinical context of DM patients, longitudinal and prospective studies of biomarkers are needed, starting at an early phase of the disease. Given the instability of most proteins in urine, careful measures to reduce pre-analytical variations are critical for reliable results.

**Fig. 3:**
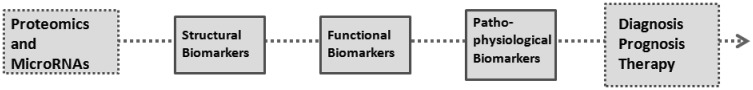
Proteomics and MicroRNAs approach to DKD

Finally, there is growing evidence for the potential of both proteomics and microRNA (miRNA) profiling to find new biomarkers for DKD. Although, it is not the aim of the present review, these two novel-approaches to discover new biomarkers are briefly considered in the context of DKD biomarker discovery. Essentially, both proteomic and miRNA approaches can explore DKD in a more dynamic and cross-sectional way that can consider structural, functional and pathophysiological pathways within the nephron as a *continuum* (Fig. 3 ).

Proteomic methods might provide a more dynamic phenotypic profile of the function and dysfunction of kidney cells, reflecting the complexity and pathophysiological changes at different stages of DKD. This methods may provide new insights into the pathogenesis of progression of DKD and might become early diagnostic biomarkers, specifically providing some functional or causative insights or associative patterns of markers (e.g. CKD273 [79]) with reliable prognostic value in the clinical setting [80], but as yet it has not been able to replace albuminuria.

It has been shown that miRNAs, noncoding RNA single-stranded molecules, are found ubiquitously in body fluids (circulating miRNAs, urinary miRNAs, miRNAs have been found also in saliva, breast milk, cerebrospinal fluid) and that their dysregulation is closely linked to altered expression of regulatory proteins in many diseases. Interestingly, they have several intriguing features that make them suitable for being considered ideal biomarkers in many clinical settings. Abundantly expressed in cells, miRNAs can be tissue- and disease-specific markers. They are more resistant to degradation than proteins and mRNA. This feature has value in kidney diseases where urine has been one of the widely used sources of biomarkers, even though it can be an unstable milieu. The high stability under extreme handling and storage conditions of miRNAs may overcome this drawback and encourage the discovery of novel urinary biomarkers in kidney disease. MicroRNAs have been demonstrated to be a useful biomarker of kidney damage in both acute and chronic situations, such as acute kidney injury, various forms of chronic kidney disease, acute allograft rejection, and in chronic allograft dysfunction [81].

Current knowledge in the context of DKD seems to show that they can reflect fibrogenesis and expansion of the mesangial extracellular matrix [82]. In a study published in 2015, Argyropoulos et al. investigated the urine profile of T1DM patients still in a normoalbuminuric phase by comparing individuals who developed signs of nephropathy over an 18-year follow-up with those who did not. The results showed that miRNA profiles were different between the two groups long before some of them evolved from normo- to microalbuminuria [83], highlighting that changes in miRNA profiles are associated with different stages of DKD. The predicted target of differentially expressed miRNAs can map to specific pathways thought to be responsible for the development of progressive DKD, e.g., collagen production, inflammation, innate immunity, toll-like receptor signalling and neovascularization, supporting data for a role of miRNAs in regulating several functions and as potential therapeutic targets. In a later study using urinary exosomes, rather than cell-free circulating miRNAs, Delić et al. found that miRNA expression was altered among T2DM patients with nephropathy compared with T2DM patients without nephropathy, and healthy controls [84]. However, the pattern of miRNAs was different compared with that found in T1DM, and neither study revealed up-regulation of miR-192 expression, one of the most well studied miRNAs in DKD associated with increased renal fibrosis.

Given the gender-related differences in miRNAs found in T1DM patients [83], the different miRNAs expressed in T1 and T2DM, and the often inconsistent results using different sources of miRNAs (i.e. renal tissue, free urinary miRNAs or urinary exosomes), further studies are needed to fully understand and optimize their potential role in clinical practice.

## Conclusion

We have explored the evidence for the contribution of the renal tubule to DKD. Diabetic tubulopathy is a real entity and although closely associated with glomerular damage, it may have a separate pathophysiology. Different metabolic and non-metabolic factors impair tubular function and probably determine some specific histological tubular changes in early and late stages of DKD. The limitations of microalbuminuria as an early and predictive biomarker of DKD still need to be overcome through the discovery and clinical evaluation of new functional or structural tubular biomarkers.

## Abbreviations

ATP: Adenosine triphosphate
AGEs: Advanced glycosylation end products
ACR: Albumin:creatinine ratio
FITC-albumin: Albumin-fluorescein isothiocyanate conjugate
ALP: Alkaline phosphatase
AT1: Angiotensin II receptor type 1
BB: Brush Border
CCL-2: Chemokine (C-C motif) ligand 2
CTGF: Connective tissue growth factor
DM: Diabetes mellitus
DKD: Diabetic kidney disease
DN: Diabetic nephropathy
ESRD: End stage renal disease
FSGS: Focal segmental glomerulosclerosis
FTB: Functional tubular biomarkers
Gpnmb: Glycoprotein non-metastatic melanoma B
GGT: γ-Glutamyltransferase
GBM: Glomerular basement membrane
GFR: Glomerular filtration rate
GSC: Glomerular sieving coefficient
GAG: Glycosaminoglycan
GLT: Glucose transporter
H-FABP: Heart fatty acid binding protein
HIF: Hypoxia-inducible factors
ICAM-1: Intercellular adhesion molecule 1
IL-1β: Interleukin 1β
IL-6: Interleukin 6
IL-8: Interleukin 8
IFTA: Interstitial fibrosis and tubular atrophy
KIM-1: Kidney injury molecule-1
KLF6: Krueppel-like factor 6
L-PGDS: Lipocalin-type prostaglandin-D synthase
L-FABP: Liver-type fatty acid binding protein
MIP-3α: Macrophage inflammatory protein-3
miRNA: MicroRNA
MAPK: Mitogen-activated protein kinase
MW: Molecular weight
MCP-1: Monocyte chemoattractant protein-1
LMWP: Proteins of low molecular weight
NAG: N-acetyl-β-D glucosaminidase
NGAL: Neutrophil gelatinase-associated lipocalin
NO: Nitric oxide
NF-κB: Nuclear factor kappa-light-chain-enhancer of activated B cells
8-oHdG: 8-Oxo-7,8-dihydro-2-deoxyguanosine
8-oxodG: 8-oxo-7,8-dihydro-2′-deoxyguanosine
P2X4 purinoreceptor: P2X4R
PKC: Protein kinase C
PT: Proximal Tubule
PTECs: Proximal Tubule Epithelial Cells
PAN: Purine Amino Nucleoside
ROS: Reactive Oxygen Species
RANTES: Regulated on activation normal T cell expressed and secreted
RRT: Renal replacement therapy
RAS: Renin-angiotensin system
RBP4: Retinol-binding protein 4
SGK-1: Serum and glucocorticoid-regulated kinase 1
STAT-1: Signal transducer and activator of transcription 1
SGLT: Sodium–glucose cotransporter
NHE: Sodium–hydrogen exchanger
STZ: Streptozocin
STB: Structural tubular biomarkers
TLR: Toll like receptor
TGF-β: Transforming growth factor beta
TBM: Tubular basement membrane
TGF: Tubuloglomerular feedback
TNF-α: Tumor necrosis factor-α
UACR: Urinary albumin-creatinine ratio
VEGF: Vascular endothelial growth factor
WT1: Wilms’ tumor-1
